# An alternative “Port”? Rationale and development process for POts Reprocessing Therapy (PORT), a brain–body therapy for postural tachycardic syndrome (POTS)

**DOI:** 10.1007/s10286-026-01189-1

**Published:** 2026-02-27

**Authors:** Taylor B Crouch, Maggie Nguyen, Mary Wells, Tammy Redman, Yoni Ashar, Howard Schubiner, Grace Westcott, Patricia Kinser, Madison Maxwell, Gisela Chelimsky, Thomas Chelimsky

**Affiliations:** 1 School of Medicine, Virginia Commonwealth University, Richmond, VA USA; 2 University of Colorado Anschutz Department of Medicine, Aurora, CO USA; 3 Ascension Providence Hospital, Southfield, MI USA; 4 Department of Psychology, Virginia Commonwealth University, Richmond, VA USA; 5 School of Nursing, Virginia Commonwealth University, Richmond, VA USA

**Keywords:** Postural tachycardia syndrome, Behavioral interventions, Mind/body approaches, Central nervous system

## Abstract

**Purpose:**

Postural tachycardia syndrome (POTS) is a chronic disorder marked by excessive orthostatic tachycardia, without clear structural/organic (e.g., cardiovascular) etiology. Recent evidence suggests that persistent autonomic symptoms may be driven by threat-induced and central nervous system-maintained dysregulation, similar to centralized chronic pain disorders. This study describes the rationale and development process of a brain–body intervention—POts Reprocessing Therapy (PORT)—aimed at reducing orthostatic symptomatology by taking advantage of the brain’s plasticity.

**Methods:**

PORT was adapted from pain reprocessing therapy (PRT), an evidence-based mind–body approach for treating centralized chronic pain. The initial protocol was developed through consultations with multidisciplinary experts, including the developers of PRT. To refine the protocol, a focus group was conducted with five women participants with POTS who underwent an early version of PORT. Rapid qualitative analysis was conducted to identify themes and inform intervention improvements.

**Results:**

The intervention consists of a medical evaluation followed by eight weekly treatment sessions. Intervention components include psychoeducation, safety learning-based exposure to symptoms, somatic inquiry, and emotional processing. Focus group participants identified reduced fear about symptoms, greater understanding of their condition, and improved functioning. Participants provided suggestions around language and content, and some shared emotional challenges of the treatment, underscoring the need for sensitive provider communication and implementation.

**Conclusion:**

This study introduces a novel therapeutic treatment for POTS that targets centrally mediated processes hypothesized to underlie persistent autonomic dysregulation. Trials are underway to formally assess feasibility, acceptability, and efficacy.

## Introduction

Postural tachycardia syndrome (POTS) is a common disorder involving the autonomic nervous system (ANS), estimated to affect 1–3 million Americans in 2019 [1, 2]. Given that viral infections are a known trigger of POTS, the disorder has become even more prevalent since the COVID-19 pandemic [3, 4]. POTS is a chronic and often disabling syndrome defined by sustained, excessive tachycardia (> 30 bpm within 10 min of standing or > 60-degree head-up tilt in adults > 20 years old) without sustained drop in blood pressure and with chronic symptoms of orthostatic intolerance while upright [5]. This definition highlights the peripheral mechanistic emphasis of nearly 30 years of studies exploring cardio- and cerebrovascular, immunologic, mast cell activation, connective tissue, and other putative mechanisms [6–8]. Beyond the definitional symptoms, patients with POTS also tend to experience physical symptoms from other comorbid disorders, including syncopal episodes, exercise intolerance, and gastrointestinal distress as well as other chronic overlapping conditions like migraine, Ehlers–Danlos syndrome, and chronic fatigue syndrome [1, 9].

### Exploring the CNS contributions to POTS

While a detailed description of POTS has emerged from past research, no study has elucidated an organic etiology in even a subset of patients. POTS has traditionally been viewed as a disorder of cardiovascular physiology, its innervation, or an immunologic attack on the same, but growing evidence on its pathophysiology suggests it may be better characterized as a disorder of the central nervous system (CNS) [10, 11]. In support of this theory, most patients with POTS experience not only cardiovascular or peripheral autonomic symptoms, but also other symptoms generally considered mediated by the CNS such as headaches, fatigue, “brain fog,” and sleep disturbance. Rates of overlap with other CNS-driven disorders (e.g., fibromyalgia, migraine, chronic fatigue syndrome) are high. In addition, neuroimaging demonstrates brain changes in areas involved in autonomic control, emotion regulation, and sensorimotor integration in patients with POTS compared to healthy controls; further, some of these brain differences such as lower insular volume have been associated with trait anxiety and depression, suggesting that brain structure is not merely an abstract anatomical finding but linked to central regulation processes [12]. Additionally, pharmacological and non-pharmacological treatments that act on the CNS, not just on peripheral management, have demonstrated benefits for POTS symptoms, suggesting that CNS dysfunction is part of pathophysiology, not just an epiphenomenon [10].

**Fig. 1:**
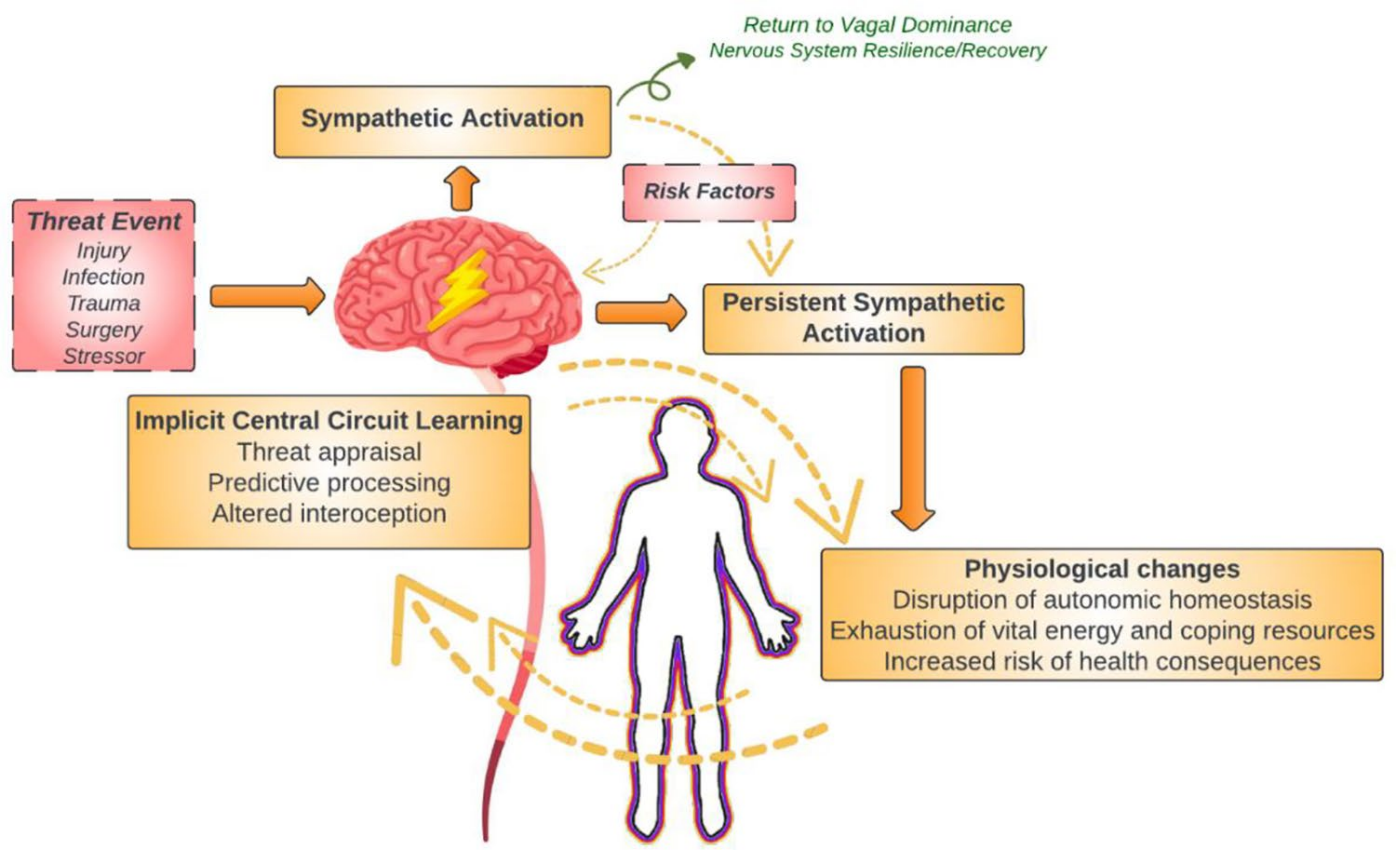
Theoretical model of central nervous system-driven autonomic dysregulation

Importantly, this model does not negate the well-established presence of structural, physiological abnormalities in POTS, such as hypovolemia, small fiber neuropathy, or altered norepinephrine levels, but rather proposes a model of interacting CNS and peripheral changes that act as part of a bidirectional feedback loop. Such interactions are not novel, as they have been demonstrated to drive other disorders such as bladder pain syndrome and irritable bowel syndrome, among many others [13–16]. In the same way, POTS may reflect a dynamic dysregulation in the interplay of complex systems (i.e., between the autonomic nervous system, cardiovascular system, and endocrine system), most notably characterized by impaired parasympathetic (vagal) regulation and persistent sympathetic activation (PSA). For example, PSA may cause the central autonomic network to fail to correctly integrate signals, which can lead to cascading effects like heart rate changes and blood pooling. Figure 1 presents an overview of this model. This integrated model combining central and peripheral mechanisms matches the clinical heterogeneity of POTS and may help explain variability in its presentations.

With this in mind, recent efforts to reclassify complex, centralized, multi-system conditions under the umbrella of functional disorders offer a compelling framework for understanding POTS. As Burton et al. (2020) propose, functional somatic disorders (FSDs) are characterized by persistent bodily symptoms maintained by dysregulated CNS processes and not fully explained by structural pathology [17]. The word “functional” has been historically controversial because such syndromes have often been forced into a false dichotomy—categorized as either “mind” or “body”; “medical” or “psychological”—which obscures the complex ways in which top-down processes can impact neurobiology and physiology. The word also carries baggage such that clinicians may misinterpret such disorders as being “in the head” or purely psychological, implying that the patient is imagining or creating symptoms, leaving the patient feeling disbelieved and in a vulnerable state. However, POTS can be described as functional such that the driving problem lies in how the ANS functions, i.e., impaired integration of sympathetic and parasympathetic (vagal) input, or in faulty communication from the nervous system to various end-organs, rather than in a structural defect or damage to the nerves or organs themselves.

Consistent with this framework, POTS shares many clinical features with chronic centralized pain conditions such as fibromyalgia, irritable bowel syndrome, and most cases of low back pain. These include symptoms often affecting multiple body systems, symptoms being triggered by innocuous stimuli (e.g., dizziness triggered by standing), symptoms that affect a body part or organ without structural damage (e.g., heart rate dysregulation despite a healthy heart), symptoms that are inconsistent (e.g., triggered by standing sometimes but not others), worsening of symptoms by stress, elevated rates of impaired functioning [18] and fear cognitions (e.g., catastrophizing [19]), and frequent overlap with sleep disturbance, fatigue, and cognitive dysfunction [20].

In chronic centralized pain conditions, the pain is considered an overlearned “alarm” response. For example, in fibromyalgia, emerging models highlight central sensitization (amplified pain processing that becomes “learned” in the CNS over time) and altered interoceptive processing as key mechanisms underlying the disorder [21]. We propose that a similar mechanism may cause and/or maintain at least some cases of POTS, wherein an initial threat activates a sympathetic response in the nervous system (likely for individuals who are already predisposed) that may become “learned” in the brain over time, or is never properly “shut off” even after the threat is removed or injury is resolved. Indeed, research indicates that POTS often develops after a threat event like an illness, infection, immunization, head trauma, psychological trauma, or surgery [22–24]. The cascading PSA likely occurs in individuals who already have some predisposition and/or other early life risk factors, though future research is needed to clarify these underlying risks as well as exacerbating factors. Symptoms like dizziness, palpitations, and fatigue in POTS may reflect not only sustained autonomic dysregulation after a threat, but also altered interoceptive prediction and threat appraisal.

Whereas the “alarm” symptom (pain) in centralized pain conditions is driven by the brain’s drive to protect the body, symptoms like dizziness, near fainting, or fainting in POTS may similarly be considered protective, built-in alarm responses of the brain, but in this case, to protect the brain itself. Fainting is a normal, reflexive response to danger, in that it occurs when blood supply or oxygen level become so low that the brain is in danger. Every person will faint if they are put on a tilt table with enough negative lower body pressure. Thus, syncope is only considered abnormal when it occurs at an inappropriate time (i.e., in the absence of a major loss of blood volume or the absence of a stimulus known to produce fainting in all subjects). By extension, POTS might also reflect an acutely salutary physiologic process designed to function in an emergency situation that continues beyond the necessary, resulting in chronic dizziness with or without fainting. While these symptoms and experiences are clearly real, we are exploring whether they may be in part driven by activation of CNS “false alarms.” Importantly, this model would consider such processes as not *consciously* generated; rather, they likely emerge from implicit predictive processes mediated by nonconscious central circuits within the brainstem and limbic–autonomic interface as has been shown in chronic pain [25].

While research has indicated that trauma exposure and post-traumatic stress disorder (PTSD) are associated with PSA and autonomic dysfunction [26], little research has examined this connection in patients with POTS. One relevant study revealed that fear conditioning may play a significant role in POTS symptoms [27]. In our descriptive cross-sectional analysis of 110 adult patients seeking treatment in our comprehensive autonomic clinic for orthostatic intolerance, POTS, or syncope [28], we found that adverse childhood experiences (ACEs) significantly predicted worse autonomic symptom severity, as measured by the Malmo Symptom Index [29], with a small effect size, such that for every additional ACE, the predicted autonomic symptom severity score increased by 2.49. Current PTSD symptomatology, as measured by a positive PTSD score on the Millon Clinical Multiaxial Inventory-IV (MCMI-IV), demonstrated a more robust positive relationship with autonomic symptom severity, along with lower global physical and mental health. While emotional traumas and/or PTSD are not present for all patients with POTS and clearly do not fully explain its etiology, these associations do lend support to the complex connection between a threat to the system and chronic autonomic dysregulation [27]. 

### Rationale for application of a neuroplasticity-based, brain–body therapy for POTS

Viewing POTS through a CNS-based, functional lens could help integrate it into broader models of chronic somatic symptoms, which may help unify understanding of overlapping syndromes. Further, it could lead to more effective treatments that directly address the underlying problem (e.g., PSA, nervous system “false alarms”) by rebalancing the system through neuroplasticity rather than addressing isolated abnormalities and symptoms. Specifically, there has been growing evidence for the use of neuroplasticity-based mind–body treatments for improving centralized pain, including pain reprocessing therapy (PRT) and emotional awareness and expression therapy (EAET), which aim to reduce or even eliminate pain by rewiring fear- and prediction-based neural pathways that lead the brain to generate pain [30]. PRT focuses on soothing CNS-generated pain by fostering mindful awareness of symptoms, intentional exposure to symptoms through a lens of safety and neutrality, reappraising symptoms as safe, and inducing positive affect to create pain reassociations in the brain. A randomized clinical trial demonstrated that participants with chronic back pain randomized to PRT had large reductions in pain intensity and disability compared to an open-label placebo injection and usual care, with many patients experiencing complete or near-complete pain relief sustained at 1-year follow-up [31]. EAET similarly targets overactive pain/threat systems, but focuses more heavily on emotional processing of traumas and interpersonal conflicts that have contributed to the threat system patterns [32].

PRT and EAET differ in important ways from traditional cognitive behavioral therapy (CBT). Traditional CBT aims to help patients improve their coping, functioning, mood, and self-management of their chronic symptoms by addressing maladaptive thoughts and behaviors, and consistent with these goals, multiple reviews and meta-analyses have demonstrated positive impacts of CBT on these outcomes, but mixed impacts on pain severity itself [33–35]. On the other hand, interventions like PRT and EAET aim to reduce physical symptoms themselves by addressing the underlying, neurobiological mechanisms that maintain the symptoms. Indeed, a recent head-to-head trial of CBT versus EAET for chronic musculoskeletal pain in older adults found that participants randomized to the EAET condition had significantly greater improvements in pain severity compared to those in the CBT condition, with improvements sustained at 6-month follow-up [36].

Here, we aim to reconceptualize POTS within this emerging paradigm, viewing autonomic dysregulation as potentially amenable to interventions aimed at restoring central homeostasis and recalibrating threat perception in the nervous system. The objective of the current study is to describe the process of developing an intervention for POTS, called POTS reprocessing therapy (PORT), which we will categorize as a *dual-focus restorative therapy (DFRT)* to emphasize that such interventions are not purely psychological, behavioral, or body-based (somatic), but rather based on the interrelationships between these brain and body processes. To do so, we utilized an iterative development process that included input from both practitioner and patient stakeholders. The current study provides an overview of this intervention development process, including qualitative feedback from patients that helped refine the intervention.

## Methods

This is a formative intervention development study that includes conceptual model building and an iterative intervention development approach, incorporating input and feedback from multidisciplinary experts and patients with lived experience with POTS. This study was approved by the Institutional Review Board at Virginia Commonwealth University.

### Expert consultation to develop intervention

The first step was soliciting and incorporating input from experts in both the target disease (POTS) and the intervention on which the treatment was based (PRT). This included interdisciplinary meetings with experts in autonomic disorders (T.Ch., G.C.), experts in pain reprocessing therapy (Y.A., H.S.), and experts in health psychology interventions broadly (T.Cr., M.W., T.R.). Based on these consultations, a draft outline with content for the intervention was developed. The structure and content were developed utilizing the PRT manual and adapting it for POTS. Specific details about the resulting intervention and how it was adapted for POTS are presented in the Results.

### Early piloting and patient focus group to refine intervention

Three health psychologists (T.Cr., M.W., T.R.) trialed an early version of the intervention with patients with POTS. The goal of this early piloting was to trial the approach and identify early challenges, successes, and needs for refinement, as opposed to close adherence to a structured protocol to systematically evaluate feasibility or efficacy. A pilot trial is now underway to evaluate feasibility, acceptability, and initial outcomes of the intervention developed and described in the current paper.

#### Participant recruitment

The medical team identified patients to undergo an early version of PORT based on the presence of POTS, per diagnosis in the medical chart, with chronic symptoms that impacted functioning. These patients were referred to one of the three health psychologists for biobehavioral treatment, which is a common clinical referral in our integrative clinic. After going through the early version of PORT, participants were contacted by a research coordinator and informed about an opportunity to participate in a one-time focus group to share their experiences. Participants were eligible for the focus group if they were over 18, had a POTS diagnosis per chart review, and had completed at least six sessions of the intervention.

#### Procedures

The focus group session was facilitated by a trained clinical research coordinator and a research assistant using a semi-structured interview protocol. Rapid qualitative analysis (RQA) procedures, which have been supported as a valid qualitative analysis approach when there are specific project goals [37], were utilized to guide qualitative data collection and analyses. This method enables efficient extraction of key themes to inform time-sensitive project goals, e.g., intervention development, while maintaining the depth of participant narratives [38]. Twelve domains of interest were identified a priori by primary researchers based on project goals, with some examples being patient perceptions of the difficulties and ambiguities encountered throughout the course of treatment, patient perception of reduction of symptom fears during treatment, evaluation of individual’s beliefs about the origins of their symptoms before and after treatment, and long-term effectiveness and sustainability of strategies learned from treatment. Open-ended interview questions were developed to reflect the domains (e.g., “What did you find challenging or confusing about the treatment?”), along with probing questions (e.g., “Tell me more about that”) that could be used as needed to clarify or expand interviewee responses. The focus group was recorded and transcribed via Zoom transcription software. The principal investigator (T.C.) and a trained qualitative research assistant (M.N.) each independently reviewed all notes taken by the interviewer and analyzed the data using the transcript and audio recording as needed, with thematic analysis and the matrix method [39, 40]. Detailed matrix summaries were compared to identify discrepancies and ensure accuracy and reliability of the thematic analysis. Discrepancies between the summaries were discussed and resolved through consensus, enhancing the validity of the findings and ensuring a comprehensive understanding of the data. The final summaries were then combined into a single, cohesive dataset for thematic analysis by T.C. and M.N. Audit trails and debriefs with the larger team were utilized to maximize rigor.

## Results

### Intervention protocol

Based on expert consultation and adaptation of the PRT intervention, we developed a treatment protocol draft that includes one medical evaluation with a medical practitioner and eight individual treatment sessions with a trained health psychologist or therapist. The protocol includes language to be used by both the medical practitioner and psychologist for psychoeducation about the role of the brain/nervous system in maintaining POTS symptoms over time, predictive processing, fainting and other symptoms as overlearned survival/protective mechanisms, and the potential improvement of symptoms from this approach.

#### Intervention steps

Patients can be referred for PORT by any of their treating medical practitioners. We developed the following general inclusion criteria for future application of PORT: (1) presence of POTS or orthostatic intolerance (upon standing, > 30 bpm heart rate increase; < 20 systolic and < 10 diastolic blood pressure decrease); (2) symptoms that are chronic (present for at least 3 months, on at least half of the days); (3) patient may have other comorbid chronic conditions (e.g., chronic pain, chronic fatigue syndrome, etc.), but POTS symptoms should be considered a primary concern; and (4) patient may have comorbid mental health conditions (e.g., anxiety, PTSD, depression) but should not have severe *and* untreated or undertreated mental illness or substance use disorder, in which case, that should be treated first.

#### Physician evaluation

After a practitioner identifies a patient who may benefit from PORT, a medical practitioner (e.g., neurologist, cardiologist) who is well versed in POTS and the current intervention approach conducts a medical assessment based on an established approach for identifying centralization of chronic symptoms [41], the goals of which are outlined in Table 1.

**Table 1:** Physician evaluation components

Goal	Process
Review patient medical history and clinical findings	Assess relevant tests (e.g., tilt table, lab results, etc.) to understand the patient’s symptoms and medical history
Rule out primary structural cause of orthostatic symptoms	Investigate potential structural causes such as genetic, cardiology (e.g., Addison’s disease), and endocrine conditions, to either rule out or ensure that these are being optimally addressed (e.g., with other current or planned medical treatments)
Assess for centralized processes contributing to symptoms	Assess history and symptoms, looking for indicators such as: (a) Presence of other co-occurring syndromes/symptoms typically related to central sensitization (b) Inconsistency in symptom presentation (c) Symptoms being triggered by innocuous stimuli; This can be assessed via patient self-report and/or with in vivo testing via imagined standing (i.e., patient closes eyes and imagines standing up, which will trigger a feeling of dizziness for some patients) (d) Linkages between stressors or trauma and onset of symptoms, both initially and ongoing *Patients are not expected to necessarily have all of the above
Provide personalized, compassionate feedback and education, elicit buy-in, prepare for treatment	Provide education about the role of the brain and POTS symptoms as manifestations of a threat response and PSA, while clearly and compassionately emphasizing that physiological symptoms are real. Emphasize expected benefits from harnessing neuroplasticity through PORT

#### Treatment sessions

The treatment protocol includes an intake with a trained health psychologist followed by eight weekly 50–60-min sessions. The primary intervention components include (1) psychoeducation; (2) gathering and reinforcement of evidence that symptoms are due to centralized processes (e.g., in-session collaborative evidence gathering, at-home diary/tracking of symptoms, optional use of a tracking/wearable device); (3) somatic inquiry, a key skill that incorporates mindfulness, messages of safety, and somatic shifting to reassociate symptoms; (4) addressing other emotional and coping threats to symptoms; and (5) gravitating more generally to positive feelings and sensations.

There are a wide variety of interventions that can be utilized to accomplish the above steps, which are detailed in the protocol. There is emphasis on experiential exercises, graded exposure to feared stimuli (physical symptoms and/or emotions), and addressing internal and external barriers to creating a sense of safety in the nervous system. Psychoeducation was adapted to focus on the ANS; e.g., sympathetic versus parasympathetic activation, the role of the vagus nerve, interactions between central and autonomic networks, differences between structural versus centrally mediated autonomic dysregulation (e.g., contrast with tachycardia caused by a heart valve disease), and the impacts of a threat or trauma. Experiential exercises were adapted to illustrate these concepts further, such as by having the patient close their eyes and imagine standing, which often triggers physiological sensations such as dizziness and/or increased heart rate. Of note, Kaufmann et al. demonstrated a similar phenomenon while patients lay supine on the tilt table prior to upright tilt [27]. Symptom exposure is a central component of the treatment in order to allow for reappraisal and real-time practice of regulation skills, which is typically achieved fairly easily with chronic pain since most patients have pain that is either constant or triggered by simple actions like sitting still or walking. In PORT, if the patient does not have chronic dizziness, imagined and/or actual standing are utilized to trigger symptoms, with emphasis on ensuring patient safety. Additionally, given the focus on retraining of the ANS, techniques for vagally mediated regulation are more heavily emphasized than in PRT—e.g., diaphragmatic breathing and other vagal stimulation techniques such as humming.

### Focus group results

The focus group consisted of five female participants diagnosed with POTS, ranging in age from 24–53. Four participants identified as White and one identified as Black. The focus group analysis identified 12 key themes reflecting participants’ perspectives on symptoms management and treatment outcomes. These themes broadly covered areas such as responses to symptoms, the effectiveness of different treatment approaches, and participants’ understanding of their symptoms. Participant quotes are included to provide further insight into these themes and to illustrate the experiences and perceptions shared during the discussion.

#### Changes in understanding of their condition from pre- to post-treatment

All participants agreed that before treatment, they experienced confusion and anxiety about the origins of their symptoms. Several struggled to reconcile their physical sensations with inconclusive or conflicting medical feedback. One participant emphasized the distress caused by contradictory explanations from different providers, which ranged from “*just anxiety and all in [her] head*” to suggestions that “*there’s really something wrong with your heart.*” Through PORT, participants reported developing a clearer understanding of their symptoms stemming from the nervous system, rather than being purely psychological or a dangerous structural defect. As one participant reflected: “*When I first started having the physical symptoms, obviously I went to like the worst-case scenario, because I was feeling the physical symptoms, and then on top of that dealing with the mental, you know, aspect of it. I really didn’t connect the two until I was speaking with [PORT clinician], and they really made me realize it, because I know that some people think that POTS isn’t considered, what do they say, functional, so the mind–body connection. However, once I realized that these symptoms I’m having are connected to my nervous system…now I see that they are completely intertwined.*” (Participant #2). Another participant shared: “*I am having physical symptoms, but there’s nothing wrong with like, my heart or my lungs. It is my nervous system causing it…I feel like she [PORT clinician], maybe even more than anything else, helped me understand what was going on in my brain and how that was affecting my body. I got a really good thorough understanding of my condition*” (Participant #5).

However, not all participants found this reconceptualization easy at first. One participant described initial resistance: “*So, my big thing was, you know, when you say the brain is causing it, it made you feel like you’re not feeling it, it’s not happening, it’s all in your head. So that was a huge thing for me that I didn’t want. I mean, I’m not crazy. I know what I’m feeling, you know. So, after understanding…and speaking with [PORT clinicians]…I could actually relate to it. So, I definitely felt that knowledge helped me.*” (Participant #3).

#### Reduction of fear during treatment

A prominent theme was the reduction of fear and anxiety associated with symptoms. All participants reported a decrease in fear over the course of treatment. While one participant described this reduction as “*moderate*,” others characterized it as “*immense*” or “*significant.*” One participant highlighted the bidirectional relationship between symptoms and fear, explaining that as her physical symptoms decreased, her fear lessened, which in turn facilitated further symptom relief. Another participant emphasized that her POTS episodes are no longer triggered by “*just sheer fear and or anxiety*.” Participants described a reduction in fear through utilizing tools provided during treatment. As one explained: “*My fear has decreased significantly using my tools, my breathing exercises, when I do feel that heightened sense of fear*” (Participant #3). Another participant described how a clearer understanding of her condition helped alleviate fear: “*Back when I first got POTS back in 2020, you know, the symptoms terrified me. It felt like I was having a heart attack with that chest pain and palpitations…What I do feel like it helped with was understanding why the symptoms were happening and just having that understanding of what’s going on in my body, I felt decreased negative feelings overall*.” (Participant #5).

#### Perceptions and evaluation of the effectiveness of somatic inquiry intervention

Participants’ evaluations of somatic inquiry were generally positive. Most participants found that somatic inquiry improved symptom experience and heightened awareness of bodily sensations. One participant described a cognitive shift in how she interpreted distressing symptoms, stating: “*I can sit there with it and just say, okay, this is normal. This is what my body is feeling like. Like I’m not, there’s nothing wrong…I’m not dying. I’m not having a heart attack. I’m not, you know, just gonna be like this forever*” (Participant #2). Similarly, a participant described using somatic tracking during physical exertion and elevated heart rate, highlighting how real-time use of the technique interrupted the fear-symptom cycle: “*I practice it a lot. I remember in the gym like when I was trying to work out and my heart rate would just get super high and, you know, I would be afraid of passing out….I would sit down and practice the somatic tracking and I noticed my heart rate coming down…so I feel like it did help me to just to center myself and be aware*” (Participant #5). Another participant noted she was already familiar with somatic tracking for managing pain and found it helped “*immensely*” with headaches, so that she was able to transfer the same strategies to her “cardiac symptoms.”

One participant shared that she initially doubted the intervention’s effectiveness, but found it more helpful after modifying her practice approach and choosing to engage in somatic inquiry during calmer states rather than when anxiety was heightened (which is encouraged in the intervention).

#### Mixed effectiveness of logging/tracking symptoms

Symptom tracking was generally perceived as a helpful tool for identifying triggers and patterns, though some participants found it overwhelming at times. However, none of the participants connected symptom tracking to its intended purpose within PORT, which is to build evidence for the centralization of symptoms, and not necessarily to reduce symptoms, “manage triggers,” or use as a long-term tool. Participants described using symptom tracking to “*see trends that influence episodes*” and “*find a lot of trends and triggers*,” particularly related to factors such as diet or menstrual cycles. One participant emphasized that while tracking did not reduce her symptoms, it helped her “*plan [her] life around it*.” Another participant highlighted both the benefits and burdens of the practice: “*There were just so many things to track, you know, what you’re eating and the migraines, the heart rate, the everything. So, a lot of the time I did feel like it was just too much. But overall, I can see how it did help and I know that. I still do track, so it is a good technique to use. Though it doesn’t help all the time*” (Participant #4).

#### Significance of the neurologist evaluation and contrast with prior medical care

Participants consistently described the neurologist evaluation as a meaningful and validating component of their care. The evaluation was seen as critical for confirming a centralized process and ruling out structural causes: “*I think that talking to a neurologist and physician was very important to me, because I was able to rule out certain conditions—that made me more, you know, like, better equipped to handle what was going on, knowing that it was just, it was POTS, like a chronic condition, as opposed to something that was structurally wrong with my body*” (Participant #1).

Participants contrasted the helpfulness of this evaluation with prior medical experiences, sharing examples of being told that symptoms were “*either caused by severe anxiety or panic attacks, or some kind of seizure*” and feeling frustrated and invalidated by disjointed care: “*All the doctors were treating everything separately*” (Participant #4). Another participant elaborated: “*Most of the doctors, maybe like, I don’t know, 90% of them, were very belittling, like, ‘nothing is wrong with you,’ you know, ‘you just need to eat more’ or, you know, ‘you need to see a therapist, you got anxiety.’ I felt like seeing everybody and running all those tests, more than anything it made my symptoms worse, because I felt so much anxiety and panic over, you know, questioning myself, like ‘is any of this real?’ It did make me worse there for a while until I saw the dysautonomia team and they started to explain everything and then things started to make sense*” (Participant #5).

#### Improvement in symptoms and functioning

Participants generally reported significant improvements in daily functioning and symptoms. Several participants described significant functional limitations prior to treatment. One reported she was passing out “*multiple times a week if not multiple times a day*,” while another shared that she could not complete basic tasks such as grocery shopping or putting her daughter in a car seat without vomiting or feeling faint. Following treatment, she described being able to actively participate in her child’s life by coaching t-ball and soccer. Some participants noted improvement in other symptoms like brain fog and sleep. One participant noted: “*I have some nights now where I’m able to sleep longer than I ever have in probably the last 20 years*” (Participant #3).

Some participants recognized that their treatment was multifaceted and that improvements may have been a result of both the intervention, medication, and other lifestyle changes. One noted, “*Everything combined together definitely gave me a new life and, you know, just to a point where my passing out [is] down to maybe once or twice a month, which is much better than it was, and I feel like I got my life back*” (Participant #5).

#### Perceptions and suggestions around language/terminology

Participants emphasized that the language used by providers can significantly shape their understanding of symptoms and influence the perceived legitimacy of their condition. Several emphasized the importance of clearly communicating the “mind–body connection,” while avoiding implications that the symptoms are “all in their head.” One participant cautioned against the potential for misunderstanding: “*You look up ‘functional’ and you see in your head and then that’s what they [people with POTS] go with and then they feel like they’re being dismissed*” (Participant #2).

Others stressed the importance of tone and delivery, especially when introducing new models that may contrast with a patient’s previous understanding: “*I think it’s the delivery of how [POTS] is being presented…it’s all in your mind and you can have control over this, at least this is how they are perceiving it…so maybe some different wording, or maybe even more of a gentle approach…you know, speaking with someone that has been going through this, you know, for 20 years…that’s kind of hard to tell someone to, you know, kind of switch over and look at it from this now, you know, like what you’ve been told is maybe not the issue*” (Participant #3).

Additionally, two participants noted that their provider did not use some of the same language shared by other participants in the focus group: “*I don’t even completely know what HRV is…*,” highlighting some inconsistencies across providers.

#### Importance of therapeutic alliance and feeling heard and validated

Participants emphasized the power of feeling heard, validated, and supported by their providers. One participant described the environment as a “*no-judgment zone*,” explaining, “*I felt like someone was listening to me, but not only listening—understanding, and not making me feel crazy*” (Participant #3). Another participant elaborated on the therapeutic value of provider communication: “*I feel like what helped me the most was really just the conversations I had with [provider] where she explained everything and listened to me and made me feel believed and validated, and I know that sounds weird. But just to have that breath of relief of being told, you know, there’s nothing wrong with you…She was like, I believe you, this is what’s going on. I felt like that was just like almost an instantaneous stop in my symptoms because I feel like I wasn’t, you know…in a constant fight or flight of trying to get people to just like take me seriously*” (Participant #5).

#### Emotional difficulties related to the treatment

Participants described the behavioral and trauma-informed approach of PORT as both empowering and, at times, emotionally challenging. A recurring theme was the tension between agency and self-blame. One participant reflected on this complexity, noting that the idea she could “control” her symptoms sometimes felt difficult to differentiate from “self-blame: “*I guess what I found challenging is the notion that I am in control of these things. I found it really liberating to actually feel like these were not my fault. Because I know they’re not my fault. I’m not like causing them or instigating them or anything like that. But I think the overarching theme of the treatment is that you can, kind of, I don’t want to use the word control, but you can increase your good times by how you react to what’s going on in your body.… So, it’s a very fine line for me. I want to believe that I can improve my symptoms. And I have been. And I can manage my condition. But at the same time, I don’t want to be told, this is within your power, because I feel like if it is within my power, then what am I doing wrong?*” (Participant #1).

Other participants shared how treatment surfaced difficult emotions tied to trauma and identity loss. One participant described grief after realizing that “*things that happened before…physical and mental trauma*” had contributed to her symptoms putting her body into a “*state of overdrive and constant fight or flight*.” Another explained the emotional challenge of “mourning [her] old life,” stating: “*That grief of knowing what happened to me is kind of permanently affecting me now…the fact that I don’t have control over it, like it happened, and this is what I’m dealing with. I’m picking up the pieces now. I feel like for me, thinking about that sometimes gets really challenging and defeating.*” (Participant #5).

Participants also highlighted the psychological adjustment involved in accepting a chronic condition without a straightforward medical solution. As one participant explained: “*I think early on, the treatment was hard. It was very difficult to accept that this is not something that, just, you can, you know, take some medication for*” (Participant #4).

#### Mixed sustainability and implementation of strategies learned from treatment

Participants generally reported continued use of key tools learned through PORT, particularly somatic tracking and breathing exercises. One participant shared that the skills had become integrated into her daily life with spontaneous use during symptom flares: “*I still do the somatic tracking. I’m actually doing the breathing as we speak, ‘cause I’m having the brain fog and…headache…*” (Participant #3).

Some noted that their engagement has decreased over time, often due to competing responsibilities, but that they modified their use. “*I still do the HRV breathing…with the device [an optional app for breathwork practice], but I overwhelmed myself…I’m like, I don’t have 20 min…so I actually do 7 min in the morning, and then depending on the evening maybe another 5 min or so*” (Participant #3). “*I do the somatic tracking and I do the breathing still, but I don’t do it as often as I should*” (Participant #5).

There was also shared recognition that not all aspects of the intervention were equally suited for long-term use. As one participant explained: “*Some of the PORT…initial stuff, like the psychoeducation and the testing everything, that’s not stuff you can keep up with, I still try to keep up with research, but that was like a one-time thing*” (Participant #5).

#### Perceptions of challenges or barriers in broader dissemination of PORT

Several participants expressed concerns that the broader POTS community might be turned off or defensive about this approach, even if it is emphasized that POTS is not a psychological condition. Patients shared experiences of invalidation or dismissal in their medical journeys, making words like “functional” or referrals to psychologists potentially triggering or off-putting. One participant explained: “*I don’t know if it’s the term functional that puts people off, and maybe they don’t completely understand that it’s not, like, a dismissal of their feelings, it’s not saying it’s all in their head. I just know that, from conversing with other people with POTS, they do feel like when you bring up this whole idea of it being functional, they kind of, almost get offended. So, I don’t know if for other people with POTS, you know, if they have that aversion to the word functional, how they would necessarily feel about PORT therapy*” (Participant #2).

Another reflected on longstanding community attitudes as a potential barrier, saying: “*I’m one of those people that has been at this for quite some years, you know, so I’m very well aware of how those in the community are looking at this, and many of them do frown upon going this route. But I think that’s because they feel that what we’ve been told for so long, you know, that it’s this, and this is what we need to do, and so many people are doing those things*” (Participant #3). Stigma within online patient communities was also noted: “*I have not had people be very receptive…I’m actually part of a Facebook group for dysautonomia and I tried sharing, just like…this is some stuff I’ve learned from my doctors…just like about the HRV and breathing, just to share, and I actually got kicked out of the group because the moderators’ comments were, you know, ‘this isn’t a psychological condition.’ It was kind of annoying because I put multiple times in the post that I knew it wasn’t just psychological…*” (Participant #5).

One participant specifically emphasized the importance of framing the intervention as a “valid medical approach” rather than a “psychiatric treatment” in order for it to be accepted. Conversely, another noted that many patients may simply be unaware of potential links between prior stressors or emotional trauma and their symptoms, suggesting that it would be very helpful for “people to see this.”

#### Patient suggestions for specific additions or modifications to the intervention

Patients recommended several improvements to the intervention. One participant emphasized the need for individualizing treatment, noting that not every component would be suitable for every patient. Multiple participants agreed that they would have appreciated more guidance around physical exercise, as they recognized the importance of moving more to reduce fear, but there was some confusion about how to approach this. As one participant noted, “*By the dysautonomia team I got told to exercise, but I was given very little information on exercising, and I feel like it would have been helpful to have been guided through it a little more…I felt like I could have used some more guidance and some more help because the gym was a very overwhelming place to be…or just exercising in general, it doesn’t have to be the gym, it could be in your own home, but exercising in general is a very stressful place to be, but it is a very important, I think, for POTS recovery and management, so more help on that would have been nice*” (Participant #5).

Additionally, consistent with the prior theme, participants suggested possibly modifying the language or delivery to avoid words that may feel invalidating or offensive to patients, and emphasizing the team approach to treating POTS. For example, one participant explained: “*If the idea of the mind–body, you know, can be explained better…or maybe the word functional not being used…So just figuring out a way, I think, to rephrase this in a way that makes sense to people still… But when I talk about working at it from all different angles as a team, it makes a little but more sense to them. I’m not sure how you could include that in PORT, but just, you know, letting people be aware that we’re not just focusing on the psychological side of it alone. We’re coming at it from all these different areas because we recognize that it’s affecting all these different things and that it’s very real*” (Participant #2).

## Discussion

This study represents an initial step toward development and evaluation of a novel conceptualization and approach to treating POTS as a functional, CNS and ANS-driven disorder that may be amenable to a dual-focus restorative therapy. We utilized an iterative intervention development process to adapt an evidence-based mind–body treatment for centralized pain, pain reprocessing therapy, for POTS. The resulting treatment—POts Reprocessing Therapy (PORT)—is based on a theoretical model that most cases of POTS likely represent a learned, neuroplastic pattern of PSA after a threat. Two stakeholder groups, interdisciplinary experts/treating practitioners and patients with lived experience who underwent an early version of PORT, were included in the development process, both of whom contributed substantially to iterative improvements.

The initial version of PORT was delivered to a small group of patients with POTS, and their feedback was elicited in a qualitative focus group to help refine the intervention. The focus group data revealed not only key insights and suggestions regarding the intervention, but also a rich picture of life with POTS before and after PORT. Participants shared common experiences prior to PORT of high fear and confusion about their symptoms, lack of clear answers from practitioners, and experiences of invalidation from medical professionals. Participants reported a notable reduction in fear associated with their physical symptoms after PORT, attributing this improvement to increased understanding of symptom mechanisms, utilization of somatic tools, and improvement in symptoms which led to further reduced fear. For many, the education and validation received from the neurologist evaluation and therapist–practitioner relationship fostered a sense of empowerment and safety, which participants described as central to their improvement. These themes, particularly those around fear reduction and the importance of the therapeutic alliance, are consistent with qualitative studies of patient experiences of pain reprocessing therapy [42].

Participants also shared challenges and complex emotional responses to the treatment approach. While many valued the autonomy and empowerment promoted by self-regulation techniques, some noted that the implication of control over symptoms at times blurred with feelings of self-blame or inadequacy. Some patients shared that it was emotionally challenging to learn about the connection between past traumas and current symptoms. Grief and identity changes emerged as salient emotional experiences, indicating that even though PORT appears to offer useful tools, it also required emotional labor and adjustment. These complexities reflect the need for strong practitioner training and nuanced clinical messaging that empowers patients without inadvertently reinforcing internalized blame. Participants also shared about the importance of language, noting stigma around the word “functional” and that they prefer language like “mind–body connection” over “psychological.”

As a result of this development process and piloting, the subsequent step has been to refine and further test the intervention. Participant feedback allowed us to make several notable improvements to the intervention, which is currently being tested in a formal feasibility trial, with a larger randomized controlled trial to follow. For example, based on the focus group data, we refined language and enhanced the practitioner guide to improve clarity and mitigate risk of invalidation. The purpose of symptom tracking—building evidence of the centralized nature of symptoms, rather than developing a burdensome or long-term monitoring practice—was more clearly defined. We incorporated specific patient recommendations, such as more guidance on physical movement with POTS. Additionally, the practitioners who delivered the early version of PORT were able to identify patterns in their treatment experiences that allowed for further refinement of the intervention. For example, therapists observed common psychosocial patterns in many patients in this population, including high self-pressure, difficulties with emotional awareness and/or expression (i.e., high levels of emotional suppression), and interpersonal challenges including difficulty with assertiveness. Each of these are now more explicitly incorporated into the module of treatment that focuses on addressing emotional and coping threats to symptoms.

## Limitations

This study was primarily focused on intervention development. The very small sample size and lack of a control group do not allow us to make any conclusions at this point about the efficacy of PORT, and further efforts are underway to systematically evaluate PORT’s feasibility and outcomes. Given that the participants in the current study received an early, in-development version of PORT, we cannot ascertain treatment fidelity or adherence, which will be monitored in future trials.

While the intent of PORT is to address the putative CNS driver for POTS, PORT would typically be combined with other treatments that address other pieces of the larger POTS picture, such as salt supplementation and graded exercise. PORT is not designed to be a standalone treatment for POTS and should ideally be integrated into a team-based approach that includes other indicated medical and behavioral interventions. PORT is not appropriate for all patients, including those who have clearly identified and untreated structural causes of their disorder. Additionally, some patients may not have an interest or willingness to participate in a mind–body treatment that involves active and sustained treatment engagement along with potential emotional adjustment challenges. It is too early to speak to dissemination potential, but we recognize that the intensive psychotherapeutic nature of the intervention may raise challenges in broadly delivering PORT.

## Conclusions

This project is part of a larger effort to develop a more holistic understanding of POTS as a disorder characterized not just by its peripheral symptomatology, but as a multidimensional condition that emerges from complex disruptions in the CNS that may be amenable to a neuroplasticity-based therapy approach. This early study focused on conceptualization and development of a brain-body treatment for POTS, which is currently being evaluated for feasibility and effectiveness. Feedback from a small sample of patients with POTS offered a promising look into the potential of this intervention to improve our understanding and treatment of POTS.

## Data Availability

De-identified data underlying the results reported in this manuscript will be made available upon reasonable request to qualified investigators, consistent with NIH data sharing policies, institutional review board approval, and participant consent.
